# Changes in Protein Levels during the Storage and Warming of Breast Milk in a Domestic Environment

**DOI:** 10.3390/children11091133

**Published:** 2024-09-18

**Authors:** Penprapa Siviroj, Jetsada Ruangsuriya, Krongporn Ongprasert

**Affiliations:** 1 Department of Community Medicine, Faculty of Medicine, Chiang Mai University, Chiang Mai 50200, Thailand; penprapa.s@cmu.ac.th; 2 Department of Biochemistry, Faculty of Medicine, Chiang Mai University, Chiang Mai 50200, Thailand; jetsada.ruang@cmu.ac.th

**Keywords:** breast milk, protein, storage, thawing, warming

## Abstract

**Background/Objectives**: Storage and warming can impact the protein concentration of breast milk (BM). This study aimed to explore the effects of routine BM handling, from storage to warming, on BM protein concentration. **Methods**: Forty BM samples were collected from 40 mothers with full-term infants. Fresh BM samples were analyzed within 24 h of expression as a baseline. Additional samples were stored in a refrigerator for up to 4 days and in either a freezer compartment of a refrigerator with a separate door (refrigerator freezers) or a deep freezer for up to 6 months. We compared four warming techniques: rapid thawing in water at 25 °C and 37 °C immediately after freezer removal, and slow thawing in a refrigerator for 12 h, followed by warming in water at 25 °C and 37 °C. The protein concentration was measured using the Lowry method. **Results**: No significant decrease in BM protein concentration was observed across all storage conditions compared to baseline. BM with a higher protein content benefited more from storage in deep freezers than in refrigerator freezers. Thawing immediately after removal from the freezer at 25 °C preserved significantly higher total protein levels compared to alternative heating techniques. **Conclusions**: Our findings support the recommendation for mothers to store BM in either type of freezer for up to 6 months. Our results suggest that rapid thawing at 25 °C is the most effective method for preserving protein levels compared to other thawing techniques used in our study.

## 1. Introduction

Breast milk (BM) protein is a crucial macronutrient that plays a vital role in supporting early human growth and development. In addition to its nutritional value, BM protein contains bioactive components that profoundly impact infant survival and health. Notably, immunologic proteins such as secretory immunoglobulin A, lactoferrin, and lysozyme provide essential immune protection, contributing significantly to the overall well-being of the infant [1,2,3]. Storage and warming can alter the protein concentration [4,5]. However, storing expressed BM remains crucial when direct breastfeeding is not feasible for infants [6,7,8,9].

Standard guidelines indicate that breast milk can remain at room temperature (25–29 °C) for up to 4 h [2,10,11,12,13]. For refrigerator storage, characterized by temperatures colder than 4 °C, the permissible storage period is extended to a maximum of 4 days [10,11,13,14]. When considering the frozen storage of BM, it is crucial to distinguish between the types of freezers and their respective operating temperatures. The freezer compartments of refrigerators with separate doors (refrigerator freezers), commonly found in most households, typically operate at temperatures below −18 °C. These refrigerator freezers allow for the storage of BM for a recommended duration of 3 to 9 months [2,10,11,12,14]. In contrast, chest or upright manual defrost freezers, often referred to as deep freezers, operate at temperatures lower than −20 °C. While these freezers may be more challenging to access, they offer the advantage of extending the storage duration of BM for up to 12 months [2,10,11,12,14]. In the past, refrigerator freezers were a common option for most households. However, recent technological advancements have led to the development of deep freezers specifically designed for domestic use. These newer models maintain temperatures below −20 °C and feature compact designs that are well suited to home environments [15]. Despite these advancements, there are limited data to guide medical professionals or mothers in deciding whether deep freezers are necessary compared to the more commonly available refrigerator freezers in general households. This gap in data points to the necessity for additional studies to establish optimal BM storage practices that effectively preserve the protein content of BM.

The final handling steps for frozen BM involve thawing and warming. International health authorities such as the Centers for Disease Control and Prevention [10], the Academy of Breastfeeding Medicine [11], and the American Academy of Pediatrics [13] recommend two common thawing methods that are practical and easy to implement in household settings. The primary method involves either placing frozen BM directly in warm water or immersing it under warm running water immediately after removal from the freezer (rapid thawing, RT). The alternative method entails placing frozen BM in the refrigerator overnight, followed by warming it under warm water (slow thawing, ST) [2,10,11,13]. However, the existing literature does not specify which thawing method better preserves the protein content of BM. In considering the optimal feeding temperature, previous research has documented the impact of milk temperature on feeding tolerance in preterm infants. For instance, Uygur et al. (2019) observed that infants fed milk warmed to 32–34 °C exhibited lower gastric residuals compared to those fed at cooler temperatures (22–24 °C), indicating better feeding tolerance at warmer temperatures [16]. Additionally, the thermal stability of proteins in BM is a critical consideration. Chang et al. (2013) demonstrated that warming BM to higher temperatures (60 °C) can degrade significant proteins, such as lactoferrin, secretory immunoglobulin A (sIgA), and leptin, better than lower temperatures (40 °C) [17]. These findings underscore the importance of selecting appropriate warming temperatures to preserve the nutritional and immunological qualities of BM, even at temperatures below those used for pasteurization. Current recommendations for feeding temperatures of BM vary, suggesting a range from cold to room temperature or warm options [10,11,18]. Despite these guidelines, specific recommendations for optimal warming temperatures at feeding times are scarce. In this study, we employed the Lowry method, a spectrophotometric technique for determining total protein content in milk and dairy products [19,20]. This method is renowned for its high sensitivity, minimal reaction interferences, and broad accessibility, making it the preferred choice for protein quantification in various samples. The principle of the assay involves the reduction of Cu^2^⁺ ions by the peptide bonds present in proteins, resulting in the formation of a blue-colored complex that exhibits absorbance at 650 nm [21].

While previous studies have examined changes in BM proteins due to various storage and thawing techniques, most have focused on pasteurized milk from milk banks [22,23,24], or utilized protocols that differ from typical domestic settings, such as storing BM at −80 °C before analysis [25,26]. Additionally, no prior studies have fully replicated the entire process of milk handling in a real-life context, including expression, storage, thawing, and warming according to standard guidelines. The aim of our study was to investigate the impact of common home storage conditions, thawing methods, and warming temperatures on BM protein levels. Our findings can provide valuable insights for policymakers, healthcare providers, researchers, and mothers, helping to determine the most appropriate equipment and techniques for managing expressed BM in home settings.

## 2. Materials and Methods

### 2.1. Subjects

We recruited 40 lactating mothers of full-term infants from four hospitals in Chiang Mai City, Thailand, between June and July 2021. These hospitals included both government and private institutions that offer secondary and tertiary care. The inclusion criteria specified mothers aged between 18 and 40 years old with full-term infants aged between 1 and 6 months. Any underlying diseases in either the mother or her infant were considered exclusion criteria. Participants were required to sign informed consent forms before providing BM samples. Details on participant recruitment and collection of milk samples have been published elsewhere [27,28].

### 2.2. Milk Collection, Storage, and Heating Procedures

**Figure 1:**
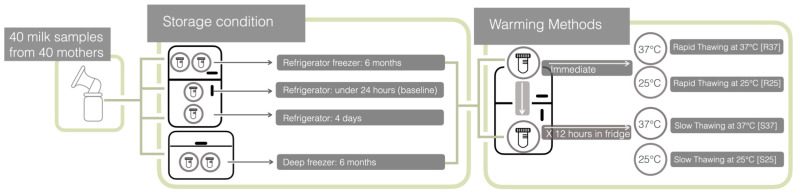
Storage and heating procedures.

The study coordinator contacted each participant 24 h before the scheduled milk collection to remind them of the appointment and recheck the inclusion and exclusion criteria. Participants were required to arrive at the lactation room at their scheduled time independently. A total of 40 mothers used Lactina Electric Selection Pumps^®^ (Medela Inc., Barr, Switzerland) between 08:00 and 11:00 a.m. Each participant used the breast pump on both breasts for at least 15 min. If no milk was expressed before the 15 min mark, participants were instructed to continue pumping for an additional five minutes after no more milk was expressed. Immediately after collection, the fresh milk from the plastic bottle was detached from the pumping set and transferred to a 50 mL polypropylene centrifuge tube (NuncTM^®^, Roskilde, Denmark). The tube was then placed in an insulated ice box for transport to the laboratory, where the milk was aliquoted into 15 mL polypropylene centrifuge tubes (NuncTM^®^, Roskilde, Denmark), each containing 10 mL of milk. Each tube was processed according to the conditions detailed in Figure 1 within four hours of collection. Baseline sample analysis was conducted within 24 h of collection. For RT, frozen samples were promptly moved from the refrigerator freezer to a water bath (Memmert Gmbl + Co., KG., Schwabach, Germany) set at either 25 °C or 37 °C for 15 min. For ST, frozen samples were transferred from the refrigerator freezer and kept in the refrigerator for 12 h. Following this, the samples were warmed in a water bath set at either 25 °C or 37 °C for 15 min (Figure 1).

### 2.3. Analytical Methods for Determining Total Protein Level

The total protein level in the BM samples was assessed using Lowry’s method with Folin–Ciocalteu solution (VWR Chemicals, cat no. 31360.264, Radnor, PA, USA). Each sample was diluted 100-fold with deionized water (DI H_2_O) before the addition of an alkaline solution and Folin–Ciocalteu solution. The mixture was incubated for 10 min at 25 °C, and absorbance was recorded at 650 nm using a Synergy H4 Hybrid Reader (Bio-Tek, Winooski, VT, USA). Bovine serum albumin (0 to 100 mg/mL; GE Healthcare, cat no. K41-001; Chicago, IL, USA) served as the standard for calculating the total protein level in each sample. The same laboratory team, consisting of three technicians and an instructor, performed the analysis each time, ensuring consistency and standardization across all processes.

### 2.4. Statistical Analysis

The data were analyzed using STATA version 16 statistical software program (Stata Corp. 2019, Stata Statistical Software: Release 16, Stata Corp. LLC, College Station, TX, USA). The normality of the continuous data was checked using the Shapiro–Wilk test. Parametric data are presented as the mean and 95% confidence interval (95% CI). To compare total protein levels, a one-way analysis of variance (ANOVA) was applied to baseline BM samples and those stored under different conditions. Additionally, comparisons were made between baseline BM samples and those subjected to various thawing techniques and warming temperatures. Pairwise comparisons were performed using Tukey’s honestly significant difference (HSD) test. Additionally, an exploratory analysis by multivariable linear regression with cluster–robust variance correction was carried out on the potential effect modification of the baseline BM protein concentration to the efficacy of deep freezing compared to normal freezing on the mean rate of BM protein concentration change at 6 months. All the statistical tests were two-sided, and significance was indicated by a *p*-value of 0.05.

## 3. Results

We analyzed the protein levels in 40 BM samples from mothers with a mean age of 29 years and a mean body mass index of 23.8 kg/m^2^. The infants’ mean age was 3.3 months.

The mean total protein levels of baseline BM, BM stored in a refrigerator for 4 days, BM frozen in a refrigerator freezer for 6 months, and BM frozen in a deep freezer for 6 months were 2.409, 2.612, 2.441, and 2.441 g/dL, respectively. The mean total protein levels for the four warming techniques, including rapid thawing by warming in water at 25 °C and 37 °C and slow thawing in water at 25 °C and 37 °C, were 2.130, 1.919, 1.782, and 1.859 g/dL, respectively (Table 1).

**Figure 2:**
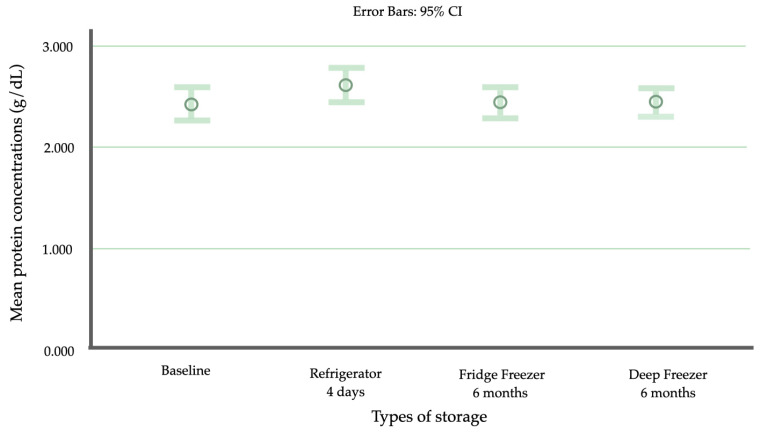
Comparison of total protein levels (g/dL) among breast milk samples stored at different temperatures for different durations. The data were analyzed using an ANOVA with Tukey’s pairwise HSD test for parametric testing. The total protein concentrations are presented as the means and the 95% CI (confidence interval).

**Figure 3:**
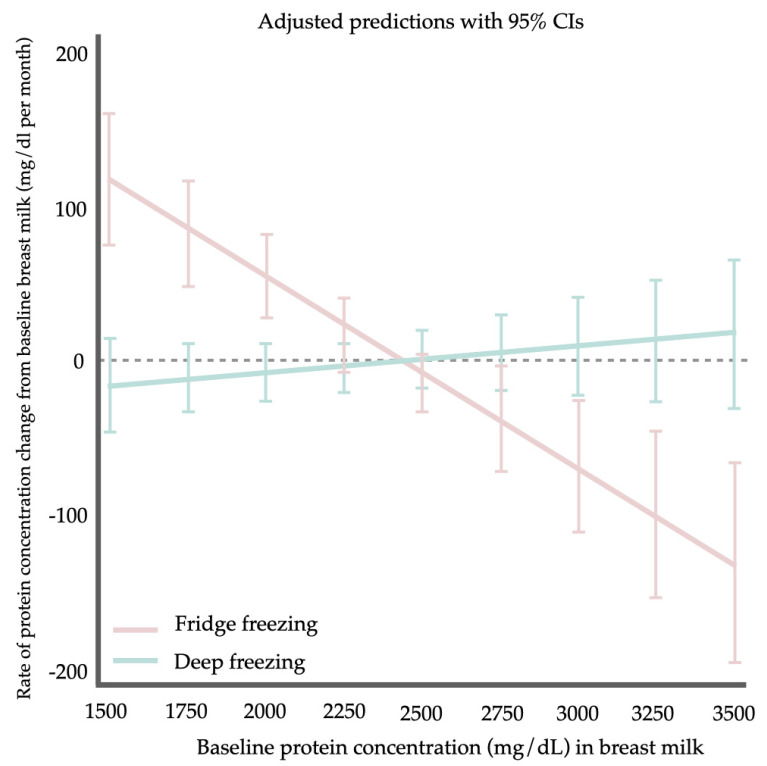
The margin plot shows the exploratory analysis by multivariable linear regression on the effect modification of the baseline BM protein concentration on the efficacy of the freezing techniques. Abbreviations: CIs, confidence intervals.

There was no significant reduction in the mean protein levels of samples stored under the three conditions when compared to the baseline (Figure 2). The exploratory analysis regarding the effect modification of the baseline BM protein concentration on the efficacy of two different freezing techniques demonstrated a higher rate of BM protein concentration decrease in the fridge freezer group over the deep freezer group by 0.139 g/dL/month per 1 g/dL of baseline BM protein concentration (95% CI 0.065–0.213, *p* < 0.001) (Figure 3).

**Figure 4:**
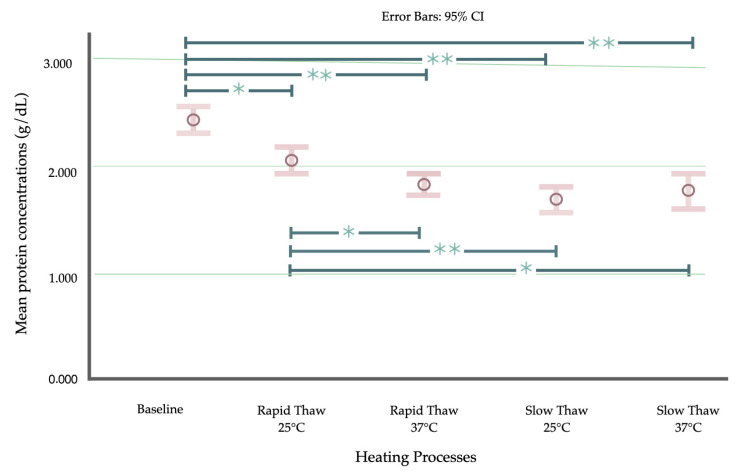
Comparison of total protein levels (g/dL) between baseline and frozen breast milk samples with different thawing conditions; rapid thawing at 25 °C; rapid thawing at 37 °C; slow thawing at 25 °C; slow thawing at 37 °C. The data were analyzed using an ANOVA with Tukey’s pairwise HSD for parametric testing. Total protein levels are presented as the mean and 95% CI; * *p* < 0.05 and ** *p* < 0.001.

The results of the comparison of the total protein levels (g/dL) between baseline BM samples and BM samples stored in a refrigerator freezer for two months under various thawing and warming conditions are presented in Figure 4. When comparing the various thawing techniques and warming temperatures, it was evident that RT at 25 °C preserved significantly higher total protein levels than alternative warming processes, including RT at 37 °C, ST at 25 °C, and ST at 37 °C.

## 4. Discussion

This study investigated the effects of various household storage conditions on the total protein level in BM. Three distinct storage conditions were explored: short-term refrigeration over a period of 4 days (temperature range: 0.8 to –5.3 °C) and long-term freezing for 6 months, both in a refrigerator freezer (temperature range: −12.2 to −18.4 °C) and a deep freezer (temperature range: −20.5 to −26.4 °C). The key findings indicate that the protein levels in BM under the three storage conditions were not significantly decreased compared to those in the baseline samples.

Despite differences in analytical methods, including protein analysis techniques, storage temperatures, and study durations, our findings are largely consistent with the existing literature. Most studies consistently demonstrate that freezing, whether in pasteurized or non-pasteurized breast milk, does not significantly reduce total protein levels, even when stored at very low temperatures (−80 °C) [24,25,26,29,30,31], as detailed in Table 2. Although protein conformation can be sensitive to changes induced by freezing [32], the temperature range used in our study, as well as in other comparable studies present in Table 2, may not reach levels that could significantly affect protein structure. Therefore, it appears reasonable to conclude that typical household storage conditions, including both refrigeration and freezing, are acceptable for maintaining protein levels in BM. Moreover, after analysis with multivariable linear regression, the baseline BM protein concentration was identified as an effect modifier impacting the efficacy of BM protein preservation between the fridge and deep freezer. BM, with a higher protein concentration, benefited more from storage in the deep freezer than in the fridge freezer. Nevertheless, a definitive conclusion about the superior efficacy of the deep freezer for preserving protein in BM cannot be made due to the small sample size. This issue needs to be explored in future studies.

Our study revealed that the RT technique was more effective in preserving total protein levels compared to the ST technique, which is consistent with the findings reported by Cao et al. in 2003. They concluded that a faster thawing rate (>10 °C/min) led to less protein damage than a slower thawing rate (<10 °C/min). This study explored the preservation of three enzymes (lactate dehydrogenase, alcohol dehydrogenase, and catalase), which are protein components in breast milk, during thawing and examined the effects of different thawing methods. The authors proposed that the changing ice–solution interface during thawing contributes to protein damage and accounts for the differences observed with varying thawing rates [34]. Additionally, our study indicates that, when comparing the same thawing method, temperatures of 25 °C and 37 °C do not show a significant difference in their effect on protein preservation in human milk. This differs from the findings reported by Chang et al. in 2013, which showed significantly greater leptin levels after thawing at 40 °C compared to thawing at 60 °C [17]. Because the degree of denaturation of milk protein at different heat-treated conditions was found to influence the outcomes related to protein preservation, the inconsistent results may be attributed to the varying protocols used, particularly the differences in study temperatures [35].

Certain limitations of this study need to be addressed. First, our analysis method, based on spectrophotometric techniques, is sensitive for protein quantification but can be affected by turbidity [19,20]. To address this, we performed a 100-fold dilution of the milk samples to mitigate potential turbidity interference and ensure precision and consistency in the results. In addition, the final concentration could be exaggerated by that kind of dilution factor. However, all samples were proceeded similarly to both the control group and the treatment samples. This processing could be normalized by the control group, especially when they are directly compared with the ratio or in the percentage of changes between/among groups. Second, we did not evaluate the cooling rate or temperature fluctuations for each storage condition. As a result, we are unable to assess the impact of these factors, which are crucial in affecting the properties of stored BM [15]. Future studies should consider investigating changes in protein properties under temperature fluctuations throughout the storage period, incorporating real-world observations of domestic refrigerators and freezers within specific regions. Particularly, previous studies have shown that household refrigerator temperatures can vary by more than 10 °C [36], with over half maintaining mean temperatures exceeding 4–6 °C, depending on the study settings [36,37]. Moreover, future studies should explore the diverse range of protein compositions in breast milk, as these proteins have varying structures and functions [1], and may be differentially affected by storage and thawing temperatures. This approach would enhance the scientific rigor and depth of subsequent research.

## 5. Conclusions

Our main finding is that the protein level of BM remained consistently stable over a 6-month storage period in both refrigerators and deep freezers. BM with a higher protein content showed more benefit from being stored in deep freezers compared to fridge freezers. RT at 25 °C was considered to be the most effective technique for maintaining protein levels in BM compared to RT at 37 °C and ST at 25 °C and 37 °C. Our findings offer insights for healthcare professionals and breastfeeding mothers. The ability to store BM for up to 6 months in either a refrigerator freezer or a deep freezer without significant loss of protein content provides a practical solution for those who cannot breastfeed directly. Furthermore, rapid thawing at 25 °C may better preserve protein levels compared to other methods, underscoring the importance of carefully monitoring temperature during the warming of BM. To optimize storage and heating protocols for breast milk (BM), future studies should explore the impact of temperature fluctuations on protein levels across varying concentrations throughout the storage period, incorporating real-world domestic storage practices. Additionally, examining a broader range of protein compositions in BM would offer deeper insights and enhance the comprehensiveness of future research.

## Figures and Tables

**Table 1:** Total protein levels (g/dL) in breast milk samples were thawed using different methods and stored at various temperatures for different durations.

Total Protein Levels	Baseline Sample	Stored in a Refrigerator (2 °C) for 4 Days	Thawing Techniques and Temperature				Frozen for 6 Months	
			Rapid		Slow		Refrigerator Freezer (–16.7 °C)	Deep Freezer (–22.3 °C)
			(25 °C)	(37 °C)	(25 °C)	(37 °C)		
Mean ± SD (95% CI)	2.409 ± 0.518 (2.243 to 2.574)	2.612 ± 0.509 (2.449 to 2.775)	2.130 ± 0.375 (2.010 to 2.250)	1.919 ± 0.288 (1.827 to 2.011)	1.782 ± 0.335 (1.675 to 1.889)	1.859 ± 0.500 (1.699 to 2.019)	2.441 ± 0.467 (2.291 to 2.590)	2.441 ± 0.411 (2.311 to 2.573)
Minimum–Maximum	1.306–3.731	1.762–3.825	1.51–3.13	1.45–2.74	1.22–2.59	1.09–3.04	1.696–4.203	1.536–3.391
Percentile (25th, 75th)	2.257 (2.097, 2.718)	2.587 (2.214, 3.004)	2.158	1.887	1.796	1.841	2.384 (2.157, 2.725)	2.424 (2.206, 2.739)

Abbreviations: CI, confidence interval; SD, standard deviation.

**Table 2:** Effect of storage and heating techniques on breast milk (BM) protein.

Year/Author	Sample Size/Type	Current LabCrop Method Protein	Baseline	Intervention			Effect
				Storage	Thawing	Warming Temperature	
2023/Binder [29]	136 samples	The Miris BM Analyser (Miris AB, Sweden)	Stored at +4 °C to +6 °C for a max. of 48 h	Freezing at −21 °C to −27 °C for a max. of 12 h	Thawing +4 °C to +6 °C over 12 h	Warmed at 40 °C	No significant change in the protein concentration between baseline and frozen samples
2020/Paulaviciene [24]	42 samples	The Miris BM Analyser (Miris AB, Sweden)	Fresh	Freezing at −40 °C for up to 10 months of (pasteurized milk (62.5 °C for 30 min))	NS	Warmed at 40 °C	Freezing and holder pasteurization did not decrease the BM protein concentration in frozen samples compared to baseline samples
2018/Paduraru [33]	90 samples (60 and 30 from mothers of preterm and full-term infants)	The Miris BM Analyser (Miris AB, Sweden)	Analyzed within 2 h after expression	Refrigerated at 4 °C for up to 72 h and frozen at −20 °C for up to 12 weeks	NS	Warmed at 40 °C	Milk frozen for more than 2 weeks contained less BM protein than milk refrigerated for up to 72 h
2016/Ahrabi [25]	40 samples	Quick Start Bradford Protein Assay (Bio-Rad Laboratories, Hercules, CA, USA)	Stored at −80 °C until analysis	Samples were removed from the freezer (−20 °C) at 1, 3, 6, and 9 months and kept at −80 °C until analyzed	NS	NS	Freezing for up to 9 months did not affect the total BM protein concentration in frozen samples compared to baseline samples
2016/Meng [23]	Pasteurized milk from 5 mothers	The bicinchoninic acid assay (PI23227; Fisher Scientific, Waltham, MA, USA).	Stored at room temperature for 12 h	Refrigerated at 4 °C for 7 days	All samples went through two freeze/thaw cycles before analysis	NS	A significant decrease in the BM protein concentration during 12 h at room temperature at 24 °C of storage (*p* = 0.02). No significant change for storage at 4 °C for 7 days
2014/Handa [26]	40 samples	A modified Quickstart Bradford Protein Assay (Bio-Rad, Hercules, CA, USA)	A baseline sample was stored at −80 °C	Stored for 7 days at −20 °C. then removed and kept at −80 °C until analyzed	1. The temperature for TW was set to 37 °C, during which the milk went through a thawing period of 10 min, followed by an additional 10 min warming phase. 2. The waterless warmer technique: Based on the quantity of milk in the container, the equipment established the time limit automatically		No differences were detected between the waterless technique and the TW technique
2010/Eduard [30]	31 samples	Lowry method	A baseline sample was analyzed as the basal control on day 0	Freezing at −20 °C for 15, 30, 60, and 90 days.	NS	NS	Freezing for up to 3 months did not affect the total BM protein concentration in frozen samples compared to baseline samples

Abbreviations: BM, breast milk; NS, not studied; TW, tepid water.

## Data Availability

The original contributions presented in the study are included in the article, further inquiries can be directed to the corresponding author.
